# Ulnar Neuropathy at the Elbow: From Ultrasound Scanning to Treatment

**DOI:** 10.3389/fneur.2021.661441

**Published:** 2021-05-14

**Authors:** Kamal Mezian, Jakub Jačisko, Radek Kaiser, Stanislav Machač, Petra Steyerová, Karolína Sobotová, Yvona Angerová, Ondřej Naňka

**Affiliations:** ^1^Department of Rehabilitation Medicine, First Faculty of Medicine, Charles University and General University Hospital, Prague, Czechia; ^2^Department of Rehabilitation and Sports Medicine, Second Faculty of Medicine, Charles University and University Hospital Motol, Prague, Czechia; ^3^Department of Neurosurgery and Neurooncology, First Faculty of Medicine, Charles University and Military University Hospital Prague, Prague, Czechia; ^4^Department of Radiology, First Faculty of Medicine, Charles University and General University Hospital, Prague, Czechia; ^5^Institute of Anatomy, First Faculty of Medicine, Charles University, Prague, Czechia

**Keywords:** ulnar nerve (MeSH), ultrasound, musculoskeletal, US-guidance, entrapment neuropathy, cubital tunnel syndrome, peripheral nerve, elbow

## Abstract

Ulnar neuropathy at the elbow (UNE) is commonly encountered in clinical practice. It results from either static or dynamic compression of the ulnar nerve. While the retroepicondylar groove and its surrounding structures are quite superficial, the use of ultrasound (US) imaging is associated with the following advantages: (1) an excellent spatial resolution allows a detailed morphological assessment of the ulnar nerve and adjacent structures, (2) dynamic imaging represents the gold standard for assessing the ulnar nerve stability in the retroepicondylar groove during flexion/extension, and (3) US guidance bears the capability of increasing the accuracy and safety of injections. This review aims to illustrate the ulnar nerve's detailed anatomy at the elbow using cadaveric images to understand better both static and dynamic imaging of the ulnar nerve around the elbow. Pathologies covering ulnar nerve instability, idiopathic cubital tunnel syndrome, space-occupying lesions (e.g., ganglion, heterotopic ossification, aberrant veins, and anconeus epitrochlearis muscle) are presented. Additionally, the authors also exemplify the scientific evidence from the literature supporting the proposition that US guidance is beneficial in injection therapy of UNE. The non-surgical management description covers activity modifications, splinting, neuromobilization/gliding exercise, and physical agents. In the operative treatment description, an emphasis is put on two commonly used approaches—*in situ* decompression and anterior transpositions.

## Introduction

Ulnar neuropathy at the elbow (UNE) represents the second most common entrapment neuropathy in the upper extremity encountered in clinical practice. The features suggesting a lesion of the ulnar nerve (UN) are based upon knowledge of the UN and its sensory and motor branch distribution. However, due to anatomic variations, a broad spectrum of differential diagnoses, and miscellaneous clinical presentations, the clinical diagnosis is often far from straightforward. If not treated timely and adequately, UNE can progress to persistent impairment of sensation, pareses, and joint contracture (1). Ultrasound (US) imaging might provide better insight into the UN morphology, mainly if the diagnosis is in doubt. The UN can be depicted using high-end US equipment with a high resolution in its course from the axilla to palm level (2). US imaging is an emerging tool in physicians' clinical practice across different specialties (3), as it allows an immediate correlation between imaging and clinical findings. It also provides a sort of “US-assisted physical examination,” e.g., “sono-Tinel” and “sono-palpation” (4). A better understanding of the relevant (sono)anatomy might help optimize clinical reasoning in patients presenting with UNE symptoms (5).

## Anatomy

**Figure 1:**
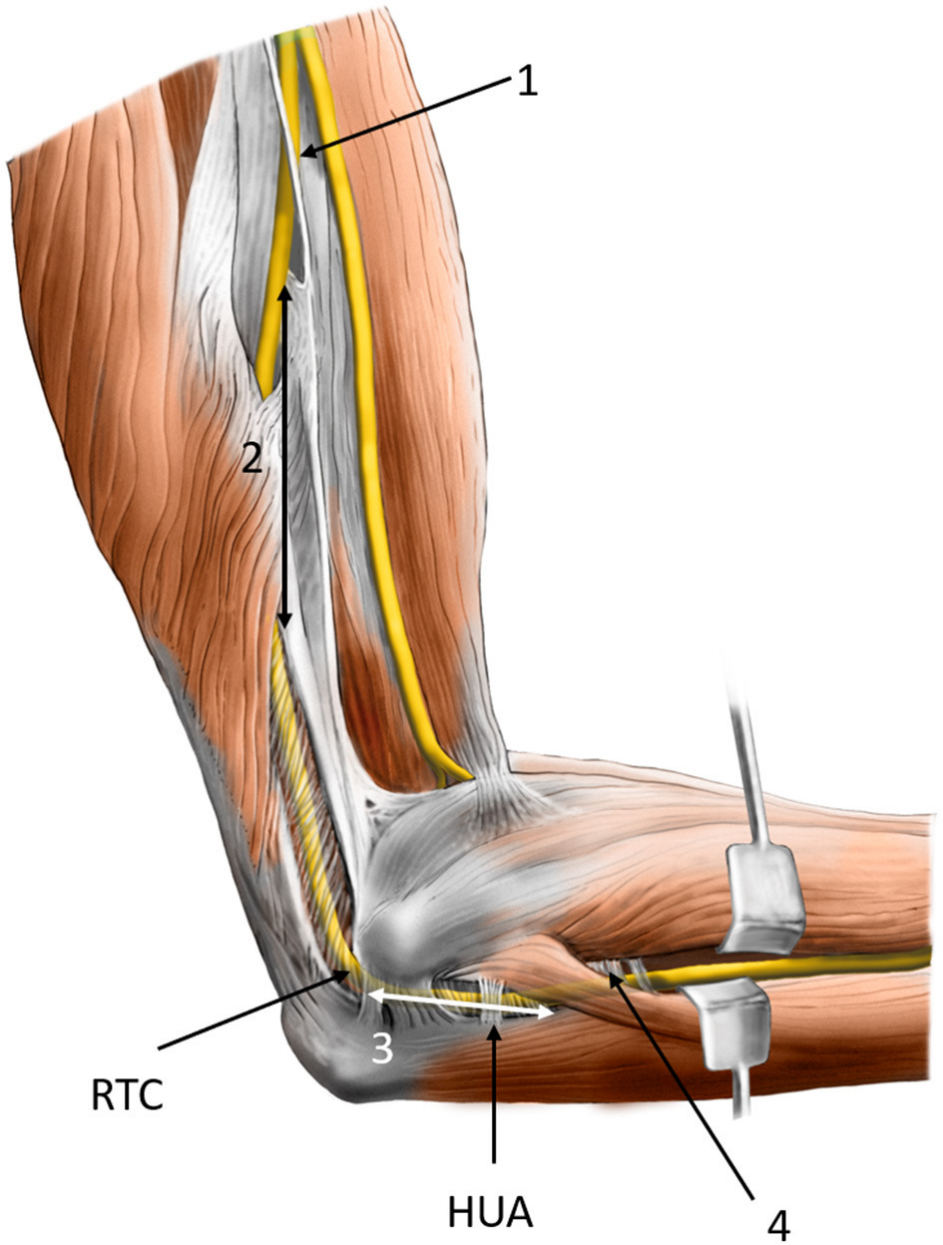
Possible sites of compression of the ulnar nerve at and around the elbow: (1) the medial intermuscular septum of the arm, (2) arcade of Struthers, (3) cubital tunnel, and (4) connective tissue between flexor carpi ulnaris and flexor digitorum superficialis muscles. RTC, retroepicondylar groove; HUA, humeroulnar aponeurotic arcade.

**Figure 2:**
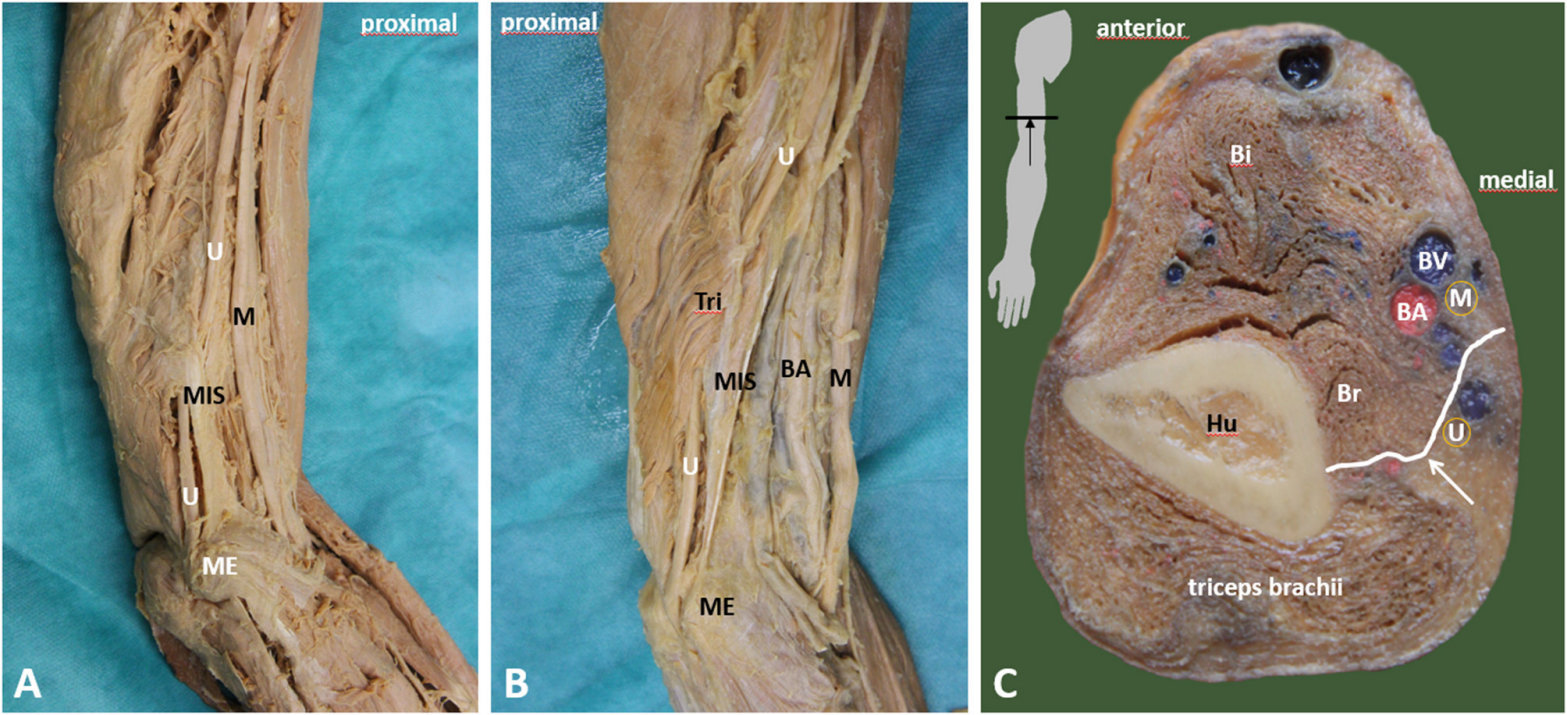
Course of the ulnar nerve in the arm. **(A)** Relationship of the ulnar nerve (*U*) and the medial septum (*MIS*). Ulnar nerve penetrates the medial septum—the penetration is demarcated by a ligamentous thickening (Struther's arcade). **(B)** Penetration of the septum and further course of the ulnar nerve is covered by muscular fibers of medial head of triceps brachii muscle, which begins on the medial intermuscular septum. **(C)** Transverse section of midarm depicting the relationship between ulnar nerve (*U*) and medial intermuscular septum (white arrow). BA, brachial artery; Bi, biceps brachii muscle; Br, brachialis muscle; BV, brachial vein; Hu, humerus; M, median nerve; ME, medial epicondyle; MIS, medial intermuscular septum; U, ulnar nerve; white arrow, medial intermuscular septum.

In practice, there are mainly two locations where the UN can be compressed: the retroepicondylar groove and under the humeroulnar aponeurotic arcade (HUA). However, the UN can be entrapped at various sites across the elbow: the medial intermuscular septum (MIS) of the arm, the thickened proximal edge of the arcade of Struthers and the entire arcade of Struthers, cubital tunnel, connective tissue between the flexor carpi ulnaris (FCU), and flexor digitorum superficialis (FDS) muscles (Figure 1). The UN is the terminal branch of the brachial plexus's medial cord and originates mainly from C8 and T1 and sometimes also receives fibers from C7 roots. At the arm level, the UN descends toward the medial bicipital sulcus along with the MIS. Approximately 10 cm above the elbow (6), the UN penetrates the MIS from the arm's anterior to the posterior compartment (Figure 2) (7). Struthers' arcade is a non-constant, morphologically variable tendinous or muscular tissue situated 6–10 cm proximal to the medial epicondyle (ME), between the medial head of the triceps brachii muscle and MIS (1). Mizia et al. (8) estimated its prevalence as 53%. Tubbs et al. (9) described three types of Struthers' arcade. Type I was described as the most common, where thickening of the brachial fascia formed the arcade. In type II, the arcade is related to the internal brachial ligament (aponeurotic continuation of the brachialis muscle), and type III arcade is due to thickened MIS (9).

In some cases, the arcade can be formed by the superficial muscle fibers of the medial head of the triceps brachii muscle as they attach the MIS (10).

Then they pass through the retroepicondylar groove (RTC, groove for the UN in formal anatomical terminology), which a floor is formed by the posterior bundle of the medial collateral ligament, and the roof is represented by a superficial fascia or non-constant retroepicondylar retinaculum. In the relaxed condition (when the elbow is extended), the retinaculum is shorter, whereas it stretches during the elbow flexion. This retinaculum was described as a structure under which UN entrapment may occur (11). O'Driscoll et al. (12) divided the retinaculum into four groups, considering its morphology and function. In type 0, the retinaculum was absent. In type Ia, the retinaculum was lax in extension and taut in full flexion not compressing the UN. Type Ib stands for the retinaculum that tights at 90–120° of flexion, with evidence of UN compression. In type II, the ligament was replaced by the anconeus epitrochlearis muscle (12).

**Figure 3:**
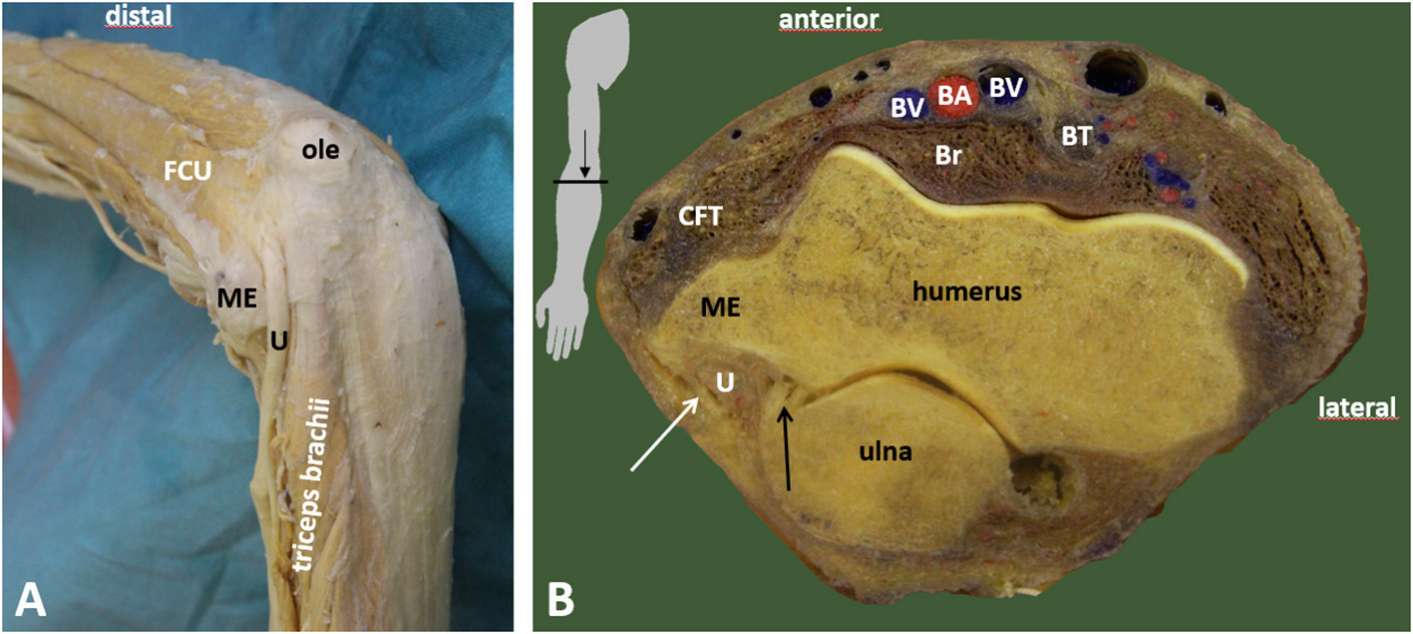
Retroepicondylar groove and the entrance to the cubital tunnel. **(A)** Entrance of the ulnar nerve to the cubital tunnel. **(B)** Demarcation of the cubital tunnel. BA, brachial artery; Br, brachialis muscle; BT, biceps brachii muscle tendon; BV, brachial vein; CFT, common flexor tendon; FCU, flexor carpi ulnaris muscle; ME, medial epicondyle; ole, olecranon; U, ulnar nerve; black arrow, medial collateral ligament, posterior bundle; white arrow, retroepicondylar retinaculum.

**Figure 4:**
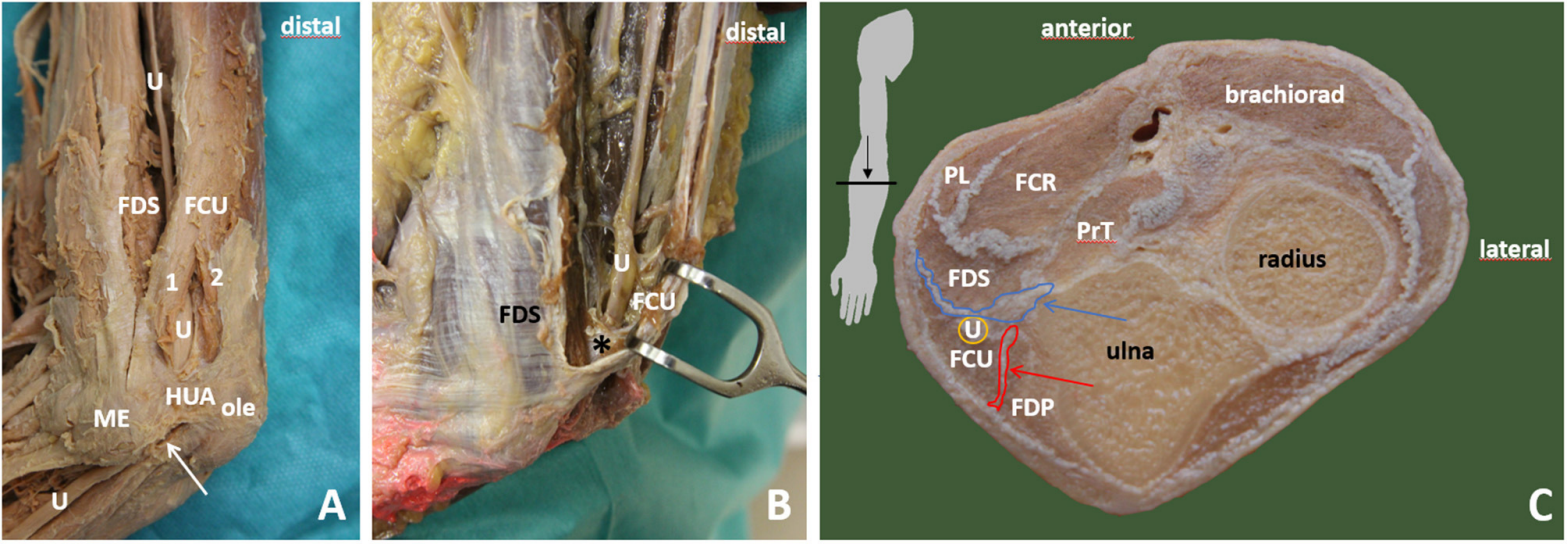
Cubital tunnel. **(A)** View on proximal part of the cubital tunnel with humeroulnar aponeurotic arcade (*HUA*) and ulnar nerve (*U*) going between humeral (1) and ulnar (2) head of flexor carpi ulnaris muscle (*FCU*). The course of ulnar nerve between FCU and flexor digitorum superficialis muscle (*FDS*). Humeral head is moved away, and the retroepicondylar retinaculum is transsected (*white arrow*). **(B)** Ulnar nerve exiting the cubital tunnel to forearm. The nerve is in close relationship to intermuscular connective tissue between FCU and FDS (*black asterisk*). **(C)** Cross section of the forearm depicting the relationship between the ulnar nerve and surrounding muscles and connective tissues (*red and blue arrows*). (1) flexor carpi ulnaris muscle, humeral head; (2) flexor carpi ulnaris muscle, ulnar head; HUA, humeroulnar aponeurotic arcade; Brachiorad, brachioradialis muscle; FCU, flexor carpi ulnaris muscle; FDP, flexor digitorum profundus muscle; FDS, flexor digitorum superficialis muscle; ole, olecranon; PrT, pronator teres; U, ulnar nerve; blue arrow, intermuscular connective tissue between FCU and FDS, deep flexor pronator aponeurosis; red arrow, intermuscular connective tissue between FCU and FDP.

The nerve continues distally behind (ME) the elbow. It enters the forearm through the true cubital tunnel (Figure 3), a space between the ulna and the ulnar and humeral heads of FCU, and a thickened fascial tissue connecting the two heads of FCU, known as the HUA (Figure 4) (13). HUA represents a thickened fascial tissue layer derived from the fusion of the antebrachial fascia and the deep fascia of the FCU (14).

After exiting the cubital tunnel, the nerve runs inside the FCU muscle and distally between the FCU and the flexor digitorum profundus (FDP) muscle. In the proximal forearm, the nerve runs at a certain distance from the ulnar artery, while more distally, the ulnar artery and nerve become adjacent. Won et al. (15) described the aponeurosis of flexor muscles of the forearm, such as intermuscular aponeuroses between the FCU and flexor digitorum superficialis, and between the FCU and the FDP as a potential site of entrapment of the UN (15).

## Epidemiology and Risk Factors

The prevalence of UNE reaches up to 5.9% in the general population (16). An increased risk for developing UNE has been reported in association with smoking (17). Another retrospective study identified increasing age and male sex as risk factors for UNE development (18). UNE development is also possible in relation to occupational hand-arm-vibration exposure (19). Interestingly, UNE was reported on the left side more frequently than on the right, regardless of the patient's handedness (20). Although recurrent subluxation or dislocation of the UN and its contribution to UNE is widely debated, some authors consider the UN instability as one of the risk factors for UNE (21). The reported prevalence of UN instability varies depending on the method of measurement. In asymptomatic arms, Van Den Berg et al. (22) reported the occurrence of UN subluxation and dislocation as 5.7 and 5.7%, respectively. According to Omejec and Podnar, the incidence rate of UN subluxation and dislocation may reach up to 27 and 20%, respectively. According to their data, the UN dislocation may cause mild damage to the UN (23).

## Pathophysiology and Causes

The UN at the elbow level can be harmed statically in entrapment neuropathies (usually below the HUA). UNE at the HUA level was reported to be associated with hard manual labor. By contrast, episodic damage to the UN may occur during specific movements (typically elbow flexion) or external compression around the retroepicondylar groove, e.g., when the forearm is lying pronated on the desk during working on the computer (a possible explanation of the more common occurrence of UNE on the left) (23). The pathophysiology of dynamic UN compression is not yet fully understood. Nevertheless, some factors associated with elbow flexion seem to play a crucial role, e.g., tightening of the retroepicondylar groove retinaculum. Furthermore, a decrease in the canal's volume, increase of intracanal pressure, and the strain of the UN accompanied by its flattening were also documented during the elbow flexion (24, 25). A congenital absence of the retroepicondylar groove retinaculum forming its roof is one of the possible explanations for the increased mobility of the UN outside the retroepicondylar groove during elbow flexion (11). Another factor possibly contributing to the UN instability would be a shallow bony retroepicondylar groove (26). However, as UN instability was reported to be common in asymptomatic volunteers, the causative relationship between symptoms and UN instability remains unclear (27). Although asymptomatic in most cases, UN instability is considered as a possible cause of pain syndrome due to friction and increased pressure applied to the UN across the ME.

Furthermore, as the hypermobile UN becomes more vulnerable during flexion, a direct trauma or pressure forces might contribute to its damage. According to Bordes et al. (28) review, the UN instability can also contribute to frictional and tractional neuritis. The concept of “frictional neuritis” assumes the subluxating/dislocating UN being irritated during the movement against bony irregularities around an arthritic or post-traumatic joint. Interestingly, Leis et al. (29) proposed complete UN dislocation as a protective factor toward the nerve strain. In entrapment neuropathy, an impaired intraneural blood flow and axoplasmic transport inside the nerve might trigger swelling. If the flow inside the nerve remains impaired, long-term intra- and extraneural fibrotic alternation with irreversible nerve damage may occur (30).

In contrast, Omejec and Podnar reported the nerve constriction as typical for UN entrapment distal to the ME by using US imaging. Simultaneously, lesions at or proximal to the ME did not show the UN's characteristic hourglass appearance, indicating its swelling in the longitudinal view (31). Other underlying causes of UNE at the elbow would comprise nerve tumors or space-occupying lesions (ganglia, accessory muscles, bony irregularities/osteophytes, or traumatic bone abruption) (32). Regarding the accessory anconeus epitrochlearis muscle, its causative role in UNE development is controversial. Wilson et al. (33) reported the occurrence of accessory anconeus epitrochlearis muscle significantly lower in patients with cubital tunnel syndrome than in asymptomatic controls. They hypothesized that anconeus epitrochlearis might be a protective factor against UNE development (33).

## Diagnosis and Ultrasound Scanning Techniques

**Figure 5:**
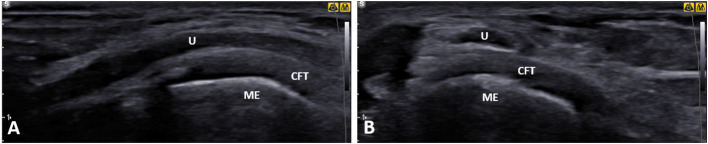
Ultrasound (*US*) imaging of the ulnar nerve (*U*) at the level of the medial epicondyle (*ME*) in a patient with symptomatic ulnar nerve dislocation. Both longitudinal **(A)** and short-axis **(B)** views clearly demonstrate a close contact between the ulnar nerve and common flexor tendon origin. U, ulnar nerve; CFT, common flexor tendon; ME, medial epicondyle.

Diagnosis is based on history, physical examination, electrophysiological assessment, and US examination. Symptoms suggesting the UNE at the elbow are medial elbow pain, tingling, and numbness in the UN supplied area (usually the fourth and fifth digits). These symptoms are commonly aggravated with elbow flexion, e.g., when talking on the phone or leaning on the elbow at the table, or sleeping with the elbow bent more than 90°. Due to neuropathic pain, sleep disturbance is common in patients presenting with cubital tunnel syndrome (34, 35). Patients sometimes describe having difficulties with typing on a keyboard, buttoning buttons, and opening bottles. However, more contributory (motor) findings suggesting UN damage are often absent initially (e.g., atrophy and weakness of the intrinsic hand muscles). The broad differential diagnosis even mounts diagnostic challenges, covering Guyon canal syndrome, carpal tunnel syndrome, C7 or C8 radiculopathy (sometimes coexisting with UNE), brachial plexopathy, or Pancoast's tumor invading its medial cord, generalized polyneuropathy, and tendinopathy (36). The UNE is often misdiagnosed as a golfer's elbow due to an intimate relationship between UN and the common flexor tendon (CFT) origin. Notably, in a case of UN instability, the nerve can be directly overlying the CFT during elbow flexion (Figure 5). In more severe cases, weakness and the UN's innervated muscle wasting can be apparent (the first dorsal interosseous muscle in particular).

Further characteristic findings of severe UNE are clawing of the ring and small fingers (also known as Duchenne's sign), Wartenberg's sign (involuntary abduction of the little finger), and a positive Froment's sign (weakening of the pinch grip between the thumb and index finger). Several diagnostic provocative tests aid in diagnosing UNE, e.g., the Tinel test at the retroepicondylar groove and the elbow flexion test with wrist extension. Additional shoulder internal rotation has been reported to increase sensitivity and specificity (24). Furthermore, impairment of two-point discrimination of the ring/small fingers can also be present. For assessing of the dislocating UN, sometimes, the nerve snapping beneath the fingertips anterior to the ME during elbow flexion can be perceived. The clinical severity is widely evaluated using McGowan's classification: Grade 1, intermittent subjective symptoms with or without mild hypoesthesia; Grade 2, remarkable sensory loss and measurable motor weakness of ulnar intrinsic hand muscles (both lumbrical and interosseous muscles); and Grade 3, persistent severe sensorimotor deficits with muscle wasting (37).

Electrodiagnosis represents a useful tool for diagnosing UNE, determining the site of entrapment and disease severity (from mild to demyelinating or axonal), aiding in prognosis, and ruling out alternative diagnoses (e.g., carpal tunnel syndrome or radiculopathy) (38). The following techniques can be used: motor nerve conduction studies (MNCSs), short segment motor studies (SSMSs), sensory nerve conduction studies (SNCSs), and needle examination. UN MNCS is a commonly performed method. As the length of the standard MNCS measured segment is 10 cm, a small lesion typical for UNE can be missed because of the dilution of the short abnormal segment in a much longer unaffected measured segment. Therefore, another method called SSMS (inching) technique is used to reveal the UN's focal damage more precisely. The elbow should be flexed to 90°, to prevent slack of the UN, which occurs when the elbow is fully extended and leads to an apparent slow conduction velocity across the elbow (39). The inching method evaluates short segments (most often 2 cm blocks) of the UN from under the elbow to above the elbow. This method's advantage is the precise localization of the nerve damage, which is important because it can influence decision making on whether conservative or surgical treatment is more beneficial (40). On the other hand, this method is technically more difficult, and despite the higher sensitivity, this method is rarely used in clinical practice. Some studies presented normative and reference values for SSMS UN evaluation (31, 41). As sensory nerves are more sensitive to compression than motor nerves, SNCS reveals pathology earlier than MNCS, but it has low significance in the diagnostic process because of its low specificity. Needle examination is important for ruling out other nerve damage sites such as wrist, brachial plexus lesion, or C8 radiculopathy. However, electrodiagnostic studies are not contributory in assessing the morphology of the UN and its surrounding tissues. A secondary cause of UN compression (e.g., ganglion and heterotopic ossification) can be missed if an imaging examination is not carried out.

Additionally, the clinical (and electrophysiological) examination can lead to an erroneous diagnosis if an anomalous innervation is present, e.g., Martin–Gruber or Marinacci anastomosis (42, 43). These forearm interconnections between the motor branches of the ulnar and median nerves account for a prevalence of up to 39% of healthy individuals (44) and can be sometimes identified with US imaging (45). To this end, US or magnetic resonance imaging should be considered, mainly if the diagnosis is in doubt. Conventional radiographs can be beneficial in assessing for the cubitus valgus, bony deformities, and space-occupying lesions (e.g., heterotopic ossification).

## Ultrasound Scanning Techniques

### Device Settings and Patient Positioning

The images and videos in this section (except for the images of exemplary pathology) were obtained using the Samsung UGEO HM70A machine (Samsung, Seoul, South Korea) with a 3–16 MHz linear transducer. Settings for the depth, gain, and frequency were adjusted by the examiner to obtain the optimal image of the UN. The focus was positioned at the same depth or just below the UN. For the comfortable UN visualization in the retroepicondylar groove, the patient is positioned supine on the examination bed. The patient's arm is resting on the examination bed with the forearm hanging over the edge of the bed, so the examiner can comfortably reach the retroepicondylar groove. The described position is comfortable for both the patient and the examiner (46). The UN evaluation and dynamic dislocation test can be easily performed, while the examination bed provides excellent probe stability. For the UN assessment at the elbow, both static and dynamic scans need to be performed (47).

### Static Evaluation

**Figure 6:**
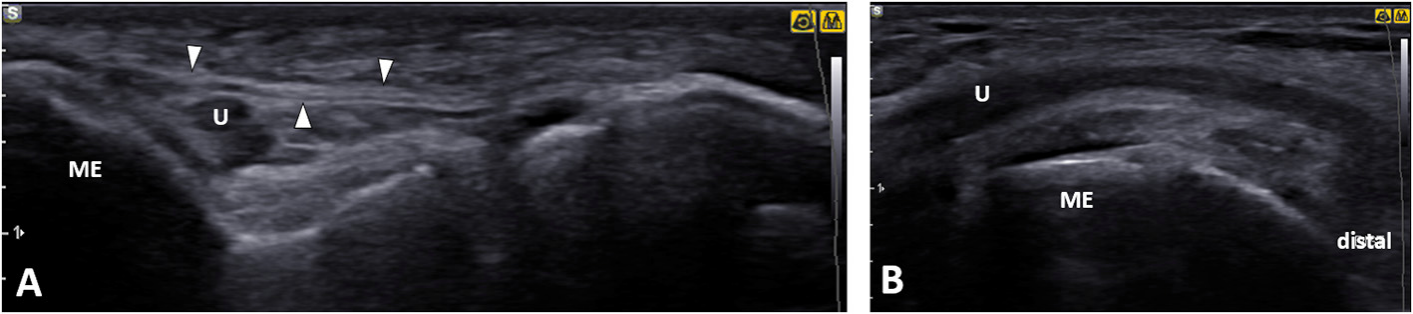
Normal ultrasound images of the ulnar nerve. **(A)** Short axis. **(B)** Long axis. U, ulnar nerve; ME, medial epicondyle; arrowheads, retroepicondylar retinaculum.

**Figure 7:**
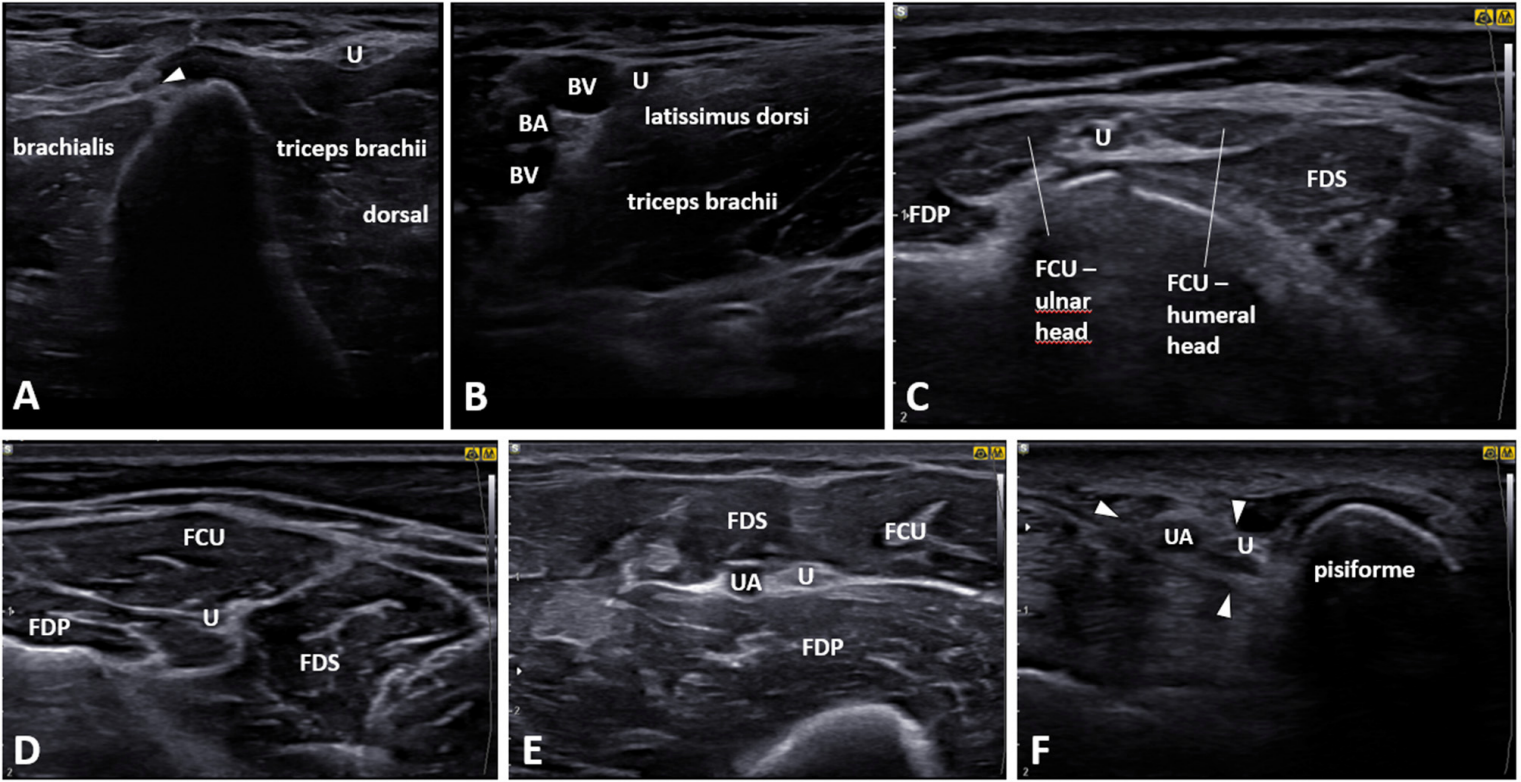
Proximal and distal tracking of the ulnar nerve starting from the retroepicondylar groove. **(A)** Midarm, arrowhead: medial intermuscular septum. **(B)** Proximal midarm. **(C)** Flexor carpi ulnaris muscle (FCU) level. **(D)** Proximal forearm. **(E)** Proximal mid-forearm, the ulnar nerve accompanied by the ulnar artery. **(F)** Ulnar nerve in Guyon canal (*arrowheads*). FDP, flexor digitorum profundus muscle; FDS, flexor digitorum superficialis muscle; U, ulnar nerve; BA, brachial artery; BV, brachial vein.

First, the transducer is positioned between the olecranon and the medial humeral epicondyle. The UN can be seen adjacent to the ME's bony surface as a uni- or multifascicular hypoechoic, round, oval, or triangle-like structure surrounded by a hyperechoic rim (Figure 6A). Due to the arching course, the UN appears hypoechoic at the retroepicondylar groove as a result of anisotropy (48). A hypoechoic band extended from the medial humeral condyle to the olecranon represents the retroepicondylar retinaculum. Rotation of US transducer 90° will change the short-axis view into a long-axis view of the UN (Figure 6B). In the authors' opinion, this is a convenient site from which the UN can be easily tracked either proximally or distally. For the proximal tracking, the UN is followed from the retroepicondylar groove further proximally. It ascends along the anterior aspect of the medial head of the triceps brachii muscle, posterior to the MIS (Figure 7A). Further proximally, at the midarm level, it inclines laterally while piercing the MIS to reach the anterior compartment, where it accompanies on the posteromedial side the proximal part of the brachial artery and brachial veins (Figure 7B). More proximally, the UN runs beside the axillary artery. For the UN distal tracking from the retroepicondylar groove level, the examiner follows the UN while entering the cubital tunnel between the humeral and ulnar heads of the FCU (Figure 7C). More distally, the UN runs inside the FCU and further between the FCU and FDP muscles (Figure 7D). In the proximal mid-forearm, the UN starts to be accompanied by the ulnar artery (Figures 4, 7E). At the wrist, the UN enters its cross-sectional triangular-shaped Guyon canal, which is superficially bounded by the palmar carpal ligament. The transverse carpal ligament forms the floor, and the pisiform represents the medial border (Figure 7F).

In general, characteristic US findings suggest nerve function impairment and swelling (usually) proximal to the compression site, loss of the normal nerve fascicular pattern, and reduced nerve mobility (47). In addition, the Doppler sonography can reveal hypervascularity to evaluate the severity of UNE (49).

**Figure 8:**
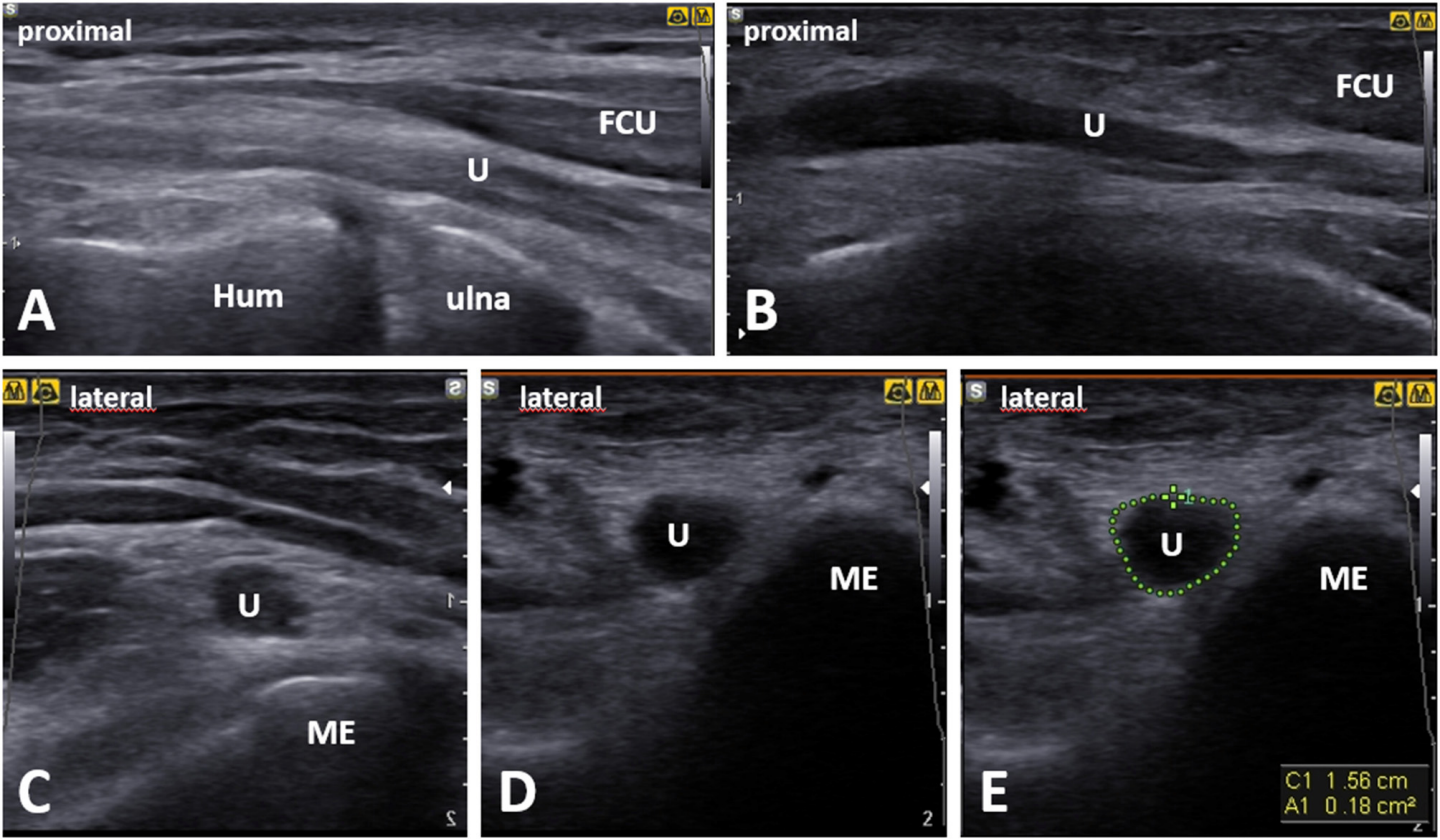
Comparative ultrasound (*US*) imaging of the ulnar nerve (*U*) at the level of the medial epicondyle (*ME*) in a patient with cubital tunnel syndrome. When compared with the normal side. **(A)** The asymptomatic side in a long axis of the ulnar nerve. **(B)** The symptomatic side ulnar nerve shows swelling (“bottle neck appearance”) proximal to the cubital tunnel inlet in long-axis. **(D,E)** In short axis, compared with the normal side **(C)**, the ulnar nerve on the symptomatic side shows enlargement in its cross-sectional area of 18 mm^2^ outlined using the direct US tracing method (*green dotted line*). Hum, humerus; FCU, flexor carpi ulnaris muscle.

An essential method to evaluate the UN statically is the measurement of its cross-sectional area (CSA) along the inner hypoechoic border (Figure 8). At the same time, the examiner can use digital tracing methods to obtain its numeric values. According to Chang et al. (50) meta-analysis, UN CSA's upper cutoff value of 10 mm^2^ at the ME level should be considered for diagnosing UNE. Mean values of 18.3 mm^2^ in CSA were reported in severe cases with axonal loss (51). As an alternative, a swelling ratio of the UN CSA_ME_/CSA_forearm_ has also been proven as a good indicator to diagnose UNE, particularly in patients with polyneuropathy (52, 53). Besides, a focal change of the UN diameter or hourglass-shaped appearance suggests of the location of the nerve lesion in case of mechanical compression or torsion (54).

### Dynamic Evaluation

While the hand of the examiner is supported on the examination bed, the patient's supine position for the UN dynamic assessment provides excellent probe stability during passive movement from extension to full flexion (usually 135°) of the elbow. Notably, the examiner should avoid too much pressure on the transducer, as this may cause deformation of the UN and prevent its dislocation. Dynamic US evaluation of the UN allows real-time visualization of the UN in high resolution throughout elbow flexion and extension. Thus, it is considered the gold standard method to assess its stability within the retroepicondylar groove. In a part of the population, the UN moves anteromedially, out of the retroepicondylar groove upon elbow flexion either onto the tip (Supplementary Video 1) or snapping entirely anterior to the ME (Supplementary Video 2). At the same time, it relocates back to its groove during extension (22). For increased mobility of the UN, Childress, in 1975, proposed a classification to type A (incomplete dislocation) and type B (complete dislocation) during elbow flexion (55). The UN hypermobility was identified in 37% and of those bilaterally in 30% as reported by Calfee et al. (56). Besides increased mobility, the UN during elbow flexion also shows a change in its shape in terms of flattening (25).

### Exemplary Pathologies

**Figure 9:**
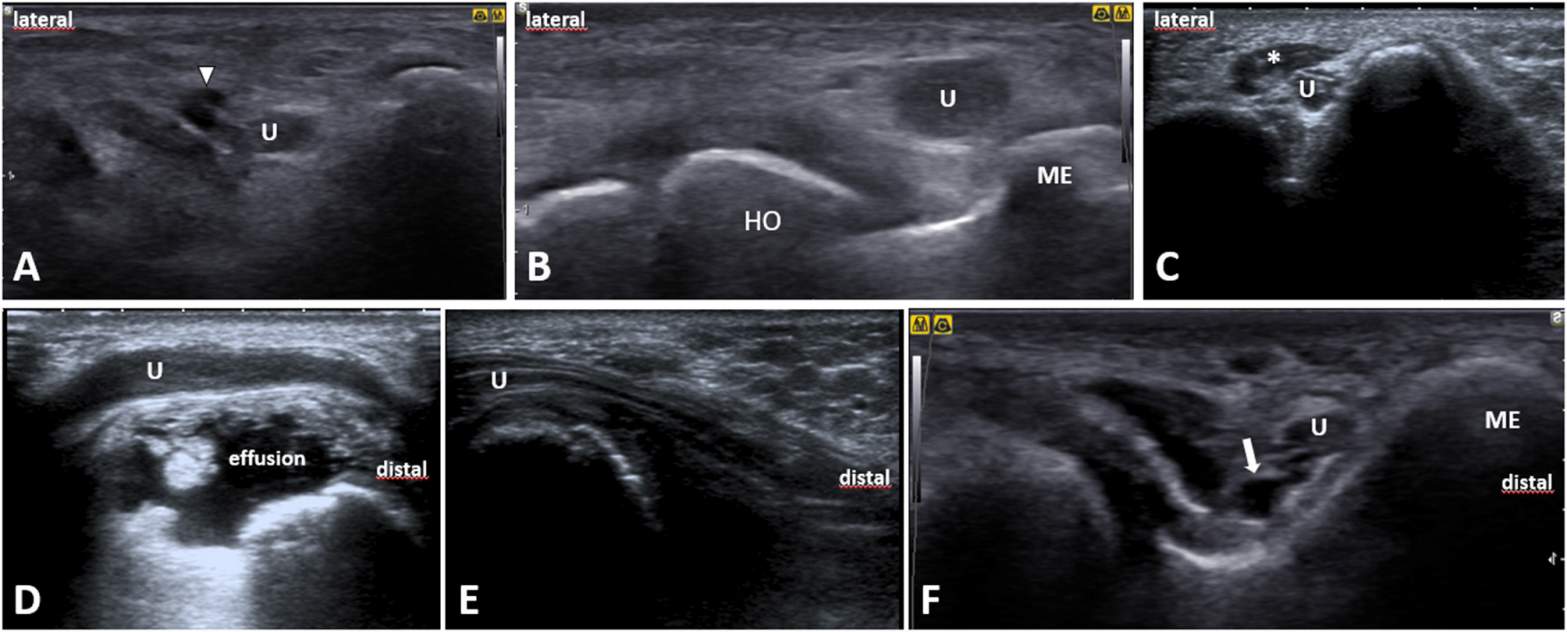
Ultrasound images of the ulnar nerve exemplary pathologies. **(A)** Short-axis image at the level of the humeral medial epicondyle (*ME*) shows the ulnar nerve (*U*) in an intimate contact with a ganglion (*white arrowhead*), likely derived from the triceps tendon. **(B)** A short-axis US image of the ulnar nerve situated just next to the heterotopic ossification (*HO*). **(C)** The ulnar nerve short-axis image shows an accessory anconeus epitrochlearis muscle (*asterisk*). **(D)** A longitudinal US image of the post-traumatic degenerative joint disease with effusion compressing the ulnar nerve. **(E)** A longitudinal image of the ulnar nerve depicts the nerve compression from scar tissue after olecranon surgery. **(F)** A short-axis view at the ulnar nerve (*U*) shows an aberrant vein (*white arrow*) next to it. ME, medial epicondyle of humerus; U, ulnar nerve.

Theoretically, the UN can be compressed at any site along its course in the upper extremity (32). Besides idiopathic entrapment neuropathy, other relevant causes of UNE are space-occupying lesions, e.g., ganglion (Figure 9A), heterotopic ossification (Figure 9B), anconeus epitrochlearis accessory muscle (Figure 9C), peripheral nerve tumors, elbow fractures associated with cubitus valgus or post-traumatic degenerative joint disease (Figure 9D), the nerve compression from scar tissue (Figure 9E), aberrant veins (Figure 9F) (57), and systemic diseases, e.g., diabetes or leprosy. Importantly, dynamic nerve irritation associated with repeated subluxation/dislocation outside the retroepicondylar groove during flexion of the elbow is also possible (Supplementary Videos 1, 2) (58).

## Non-surgical Treatment

Conservative treatment of UNE region mainly consists of approaches based on empirical experience more than on a significant level of quantified evidence. A key component of the treatment is to instruct the patient concerning risky arm positions, along with situations and movements that should be avoided. Furthermore, non-operative treatment often includes anti-inflammatory medications, manual therapy, splinting, kinesiotaping, exercise and neurodynamic mobilization, electrotherapy, shock wave therapy, dry needling, and injections. In general, the non-surgical treatment seems to be less suitable for patients with persistent post-traumatic cubital tunnel symptoms (59). Omejec and Podnar reported a study on 96 patients where the treatment was tailored based on the presumed mechanism of the UN's compression. The patients with external compression were instructed to avoid risky positioning, and those with entrapment under the HUA were offered surgical release. They reported an improvement in 83% of HUA and 84% of RTC patients. In line with this strategy, another 11 patients who were treated contrary to their recommendations showed less favorable outcomes (60).

The majority of studies on conservative treatment of UNE consists of case reports or case series with a low number of patients. Nearly all studies demonstrated clinical improvement in patient symptoms over time. However, the absence of adequate controls made it difficult to distinguish the natural amelioration of cubital tunnel syndrome from the effects of therapy (61).

The latest Cochrane review on the treatment of UNE identified only two studies on the treatment of UNE using conservative approaches (62). Besides, it was not very clear when to treat a person with this condition conservatively or surgically (62). Another recent systematic review confirms the paucity of literature and high-quality studies regarding the conservative management of cubital tunnel syndrome. The following treatment modalities were identified: education and activity modification, splinting, steroid/lidocaine injection, nerve mobilization/gliding, pulsed US, laser therapy, non-steroidal anti-inflammatory drugs, and physiotherapy. Kooner et al. (61) systematic review suggested that activity modification/education and splinting may be effective for mild or moderate disease.

Svernlöv et al. (63) published one of the few clinical trials evaluating the conservative treatment of cubital tunnel syndrome. This study of 3 months' duration enrolled 70 subjects with mild-to-moderate discomfort, while 51 subjects completed the study. All patients were employed as manual laborers. The subjects were randomly divided into three groups. One group was instructed to use a prefabricated elbow brace each night for 3 months. The brace prevented flexion of more than 45°. The second group was instructed to perform nerve gliding exercises. The third group did not perform exercises or apply any night braces. All three groups received the same written information on the anatomy of the UN, an explanation of the probable pathomechanics, and a regimen regarding the avoidance of movements and positions provoking the symptoms. Surprisingly, after 6 months, there was no significant difference in hand function, pain, strength, and neurophysiological examination. Ninety percent of patients with mild-to-moderate cubital tunnel symptoms (most patients had normal electrodiagnostic testing) improved with non-surgical treatment. In that study, 10% of patients had proceeded to surgical intervention at 6 months. Information on the causes of the condition and how to avoid provocation appeared sufficient, while night splints and nerve gliding exercises did not add favorably in this patient group (63).

### Instructions to the Patients

It is supposed that traction is one of the key mechanisms causing harm to the UN, while an elevated level of strain is strongly associated with elbow flexion. Furthermore, the duration of abnormal postures or repetitive motion probably plays a significant role in the UNE development. The strain in UN is particularly increased when nerve gliding is limited. As Vinitpairot et al. (64) described on a cadaveric model, the strain on the UN can increase if nerve gliding is restricted by 154% while working on a computer. The long-term static activity of the FCU muscle, e.g., when using a cell phone or working on a computer, or in relation to some occupations (e.g., glassmakers), probably also plays an important role. If repetitive external pressure and traction occur, often concerning activities that provoke pain and paresthesia, these symptom-causing activities should be avoided or modified. The importance of modification of movement regime was demonstrated in the above-mentioned study by Svernlöv et al. (63), where night splints and nerve gliding exercises did not add any benefit in addition to the simple instruction to avoid provocative moments (63). Arm position control may be difficult during sleep when the arm may move into a sharp flexion of the elbow beyond conscious control; hence, the use of a night brace may be appropriate in some cases.

### Splinting

The main principle of splinting is the reduction of compressive and tensile pressure on the UN by limiting elbow flexion (65). A nightly fixation of the elbow with a splint made of plastic material with good padding from the middle of the upper arm all way to the hand (30–35° flexion of the elbow, forearm at 10–20° pronation, and the wrist in a neutral position) for 6 months led to a significant amelioration of symptoms (66, 67). Nocturnal splinting can be shorter in clinical practice than the 6 months mentioned above, depending on symptom relief. Other splint options range from rolled towels placed in the antecubital fossa and secured with an elastic bandage using a neoprene brace with aluminum reinforcement to rigid thermoplastic custom-fit orthoses.

### Neuromobilization/Gliding Exercise

Therapeutic approaches based on neurodynamics have become a popular model for manual therapeutic techniques in peripheral nerve neuropathy. In particular, Butler's description of these techniques has become the norm (68). A fundamental premise of this concept is that intraneural swelling at the affected peripheral nerve site restricts intraneural blood flow (69). Simultaneously, correctly applied dynamic changes in intraneural pressure can act in a “pumping action” or “milking effect” and thus reduce this intraneural swelling together with a reduction of the symptoms (70, 71). Another assumption is that neurodynamic techniques may limit fibroblastic activity and minimize scar formation via normal and early use of mesoneurial gliding tissues (72).

The basis of this therapeutic concept is two different techniques—a sliding technique and a tensioning technique. Generally speaking, sliding is achieved by increasing the tension on the peripheral nerve by correctly applying changes in joint position at one end and releasing the tension of the nerve at its opposite end—in the UN, this is elbow flexion and simultaneous shoulder abduction or vice versa. Tensioning is achieved by increasing the tension of the nerve at both ends at one time. Indeed, in cadavers, it has been shown that a typical UN sliding technique does cause nerve movements of 8.3 mm proximal to the elbow with almost no impact on the nerve strain while tensioning causes a nerve displacement of only 3.8 mm and stretches the nerve by 9.8%. From these data, it seems that the sliding technique is less aggressive and may be more appropriate for acute injury, postoperative management, and situations leading to nerve irritation and entrapment such as bleeding and inflammation around the nerve (73). However, while in the case of carpal tunnel syndrome, neural mobilization showed some positive neurophysiological effects (e.g., reduced intraneural edema), the effect on cubital tunnel syndrome remains uncertain (74). However, it should be emphasized that the successful use of neurodynamic techniques depends, of course, on the experience and skills of the physiotherapist or physician and their ability to correctly implement these techniques in patients and to combine these approaches with manual soft tissue release (fascias in particular), forearm muscle relaxation (especially FCU muscle), and other manual techniques.

### Electrotherapy, Shock Wave Therapy, and Laser Therapy

As in the case of electrotherapy, shock wave therapy, or laser therapy in the treatment of UNE, there is insufficient evidence for a clear choice of an effective approach. Bilgin Badur et al. (75) published one of the few double-blind, randomized controlled clinical trials. In this study, the authors evaluated the therapeutic effect of shortwave diathermy in the treatment of UNE. Sixty-one patients completed the study, while approximately half of them (*n* = 31), randomly selected, were treated using shortwave diathermy 10 times over 2 weeks. The control group was given a placebo shortwave diathermy. Both groups were given elbow splints and instructed to avoid activities likely to provoke symptoms. Three months after the intervention, there was no significant difference between the groups regarding health status as measured by SF-36 (short form) questionnaires, pain, or hand function (75).

In clinical practice, the use of shock waves is widespread across the world in patients with different diagnoses. The presumed effect of the shock wave on the peripheral nerves is based on animal studies using a rat model (76, 77). The shock wave's effectiveness in patients with other types of entrapment syndromes, especially carpal tunnel syndrome, has previously been studied. Compared with the application of therapeutic US, patients with carpal tunnel syndrome who received extracorporeal shock wave therapy showed a more significant improvement in pain and hand function parameters at 12-week follow-up (78). In another randomized clinical trial, Raissi et al. (79) showed a comparable clinical outcome in patients with carpal tunnel syndrome treated with (1) wrist splints alone and (2) wrist splints + extracorporeal shock wave therapy. However, in the group with added shock wave therapy, a more favorable effect was demonstrated in median nerve distal sensory latency in nerve conduction studies (79). These results were in line with a recently published study by Gesselbauer et al., (80) who found promising clinical and electromyography (EMG) improvement after three sessions of focused extracorporeal shock wave therapy in patients with mild-to-moderate carpal tunnel syndrome and no improvement in the control group. Notably, a pilot study evaluating the effect of extracorporeal shock wave therapy for cubital tunnel syndrome has also been presented (81). Seven patients (10 elbows) received three radial extracorporeal shock wave sessions (2.000 shots, 4 bar, 5 Hz) in a total period of 3 weeks. As assessed by the Quick DASH questionnaire, the upper limb function showed significant improvement at all follow-up points evaluated within 12 weeks after therapy. According to the visual analog scale (VAS), the pain assessed was also significantly reduced (mean decrease from 4.7 ± 0.3 to 2.2 ± 0.2). The most significant improvement was in the first month after treatment. No placebo group was included in this pilot study. Nevertheless, the mean symptom duration in this study was 27.9 months, and spontaneous remission of symptoms in this patient group was not very likely. Other potential treatment options for UNE include low-laser therapy. Ozkan et al. (82) showed promising results of this therapy on functional, clinical, and electrophysiological outcomes. All beneficial effects lasted, in contrast to the US-treated group, until the third month of follow-up. Nevertheless, there was no control group in this study (82).

### Priessnitz's Wrap

To the best of our knowledge, there is no study evaluating the effect of Priessnitz's wrap on the effectiveness of UNE therapy. However, our clinical experience with this treatment is favorable. Priessnitz's wrap consists of applying two layers to the elbow area: (1) a wet squeezed cloth is applied directly to the skin, and (2) the second layer is a dry cloth serving as thermal isolation. In approximately the first 15 min, application of this wrap causes tissue cooling, followed by local hyperemia. The duration of the described wrap can range from several dozens of minutes to several hours. The assumed effect is mainly against swelling along with anti-inflammatory action. The CSA of the UN, as measured sonographically, is expected to be reduced after several applications. However, there is no published evidence for this assumption at this time, and this is only an expert opinion of the authors of this paper.

### Dry Needling

Anandkumar and Manivasagam reported three cases of patients with confirmed cubital tunnel syndrome. All patients had previously undergone unsuccessful treatments, including medication, massage, exercise therapy, US therapy, neurodynamic mobilization, and taping. The patients were treated four times over 2 weeks with dry needling, targeting the FCU muscle in two patients. In one patient, the needle was superficially inserted between the ME and the olecranon process. At discharge at 6-month follow-up, all three patients were pain-free and fully functional (83). Of note, to minimize possible adverse effects of nerve damage during the dry needling procedure, sonographic monitoring is advantageous.

### Ultrasound-Guided Injection Techniques and Exemplary Evidence

**Figure 10:**
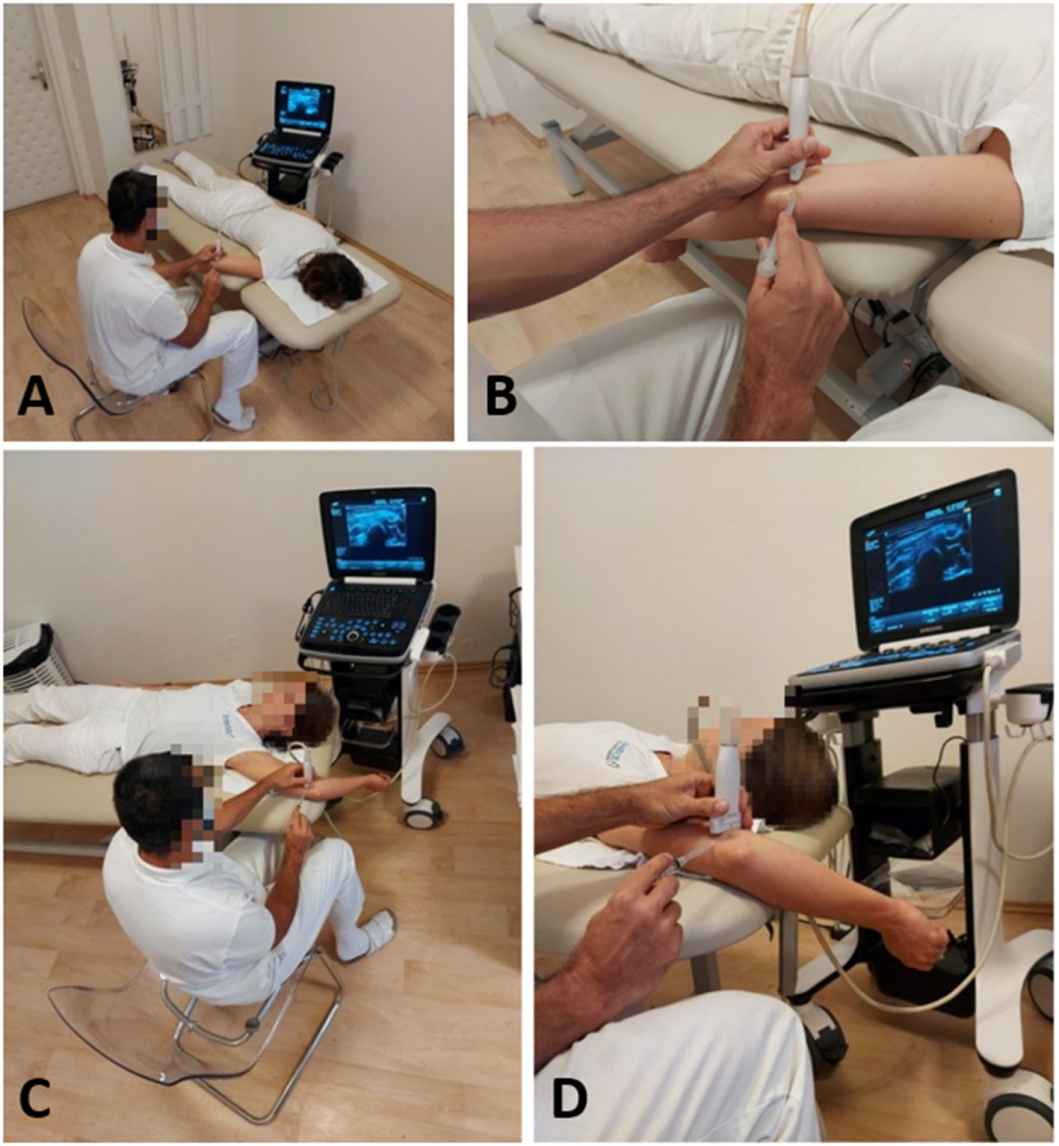
**(A)** Prone position for the ultrasound-guided ulnar nerve in-plane injection with the elbow flexed and hanging over the examination bed. **(B)** The same procedure as described before in detail. **(C)** Supine position for the ultrasound-guided ulnar nerve in-plane injection; the patient is positioned on the examination bed with the elbow flexed and hand over the head. **(D)** The same procedure as described before in details.

The patient's position is either lying supine with the elbow flexed and hand over the head (Figures 10C,D) or lying prone with the elbow bent and hand hanging over the examination bed (Figures 10A,B). As the UN at the elbow is close to the skin surface, a high-frequency linear transducer can be effectively used. Vascular structures and local abnormalities should be clarified in advance when planning the needle trajectory (84). Rules of the standard aseptic technique should be followed. Before the injection itself, a basic evaluation of the nerve and surrounding structures should be performed. A thin, e.g., 25-gauge, needle is usually preferred. The injected volume varies from 2 to 5 ml. A combination of steroids and a local anesthetic is commonly administered (85).

The UN should be visualized in the short axis, while the in-plane approach can be used. This technique allows constant visualization of the nerve's margins and the needle tip during the procedure. This technique showed a lower risk of intraneural application of the injectate (86). According to Kim and Choi, the needle should be inserted into the cubital tunnel at the ME level penetrating the retroepicondylar retinaculum (87, 88). The needle tip should be placed tightly adjacent to the nerve between the ME and the UN. To prevent compartment syndrome with persistent paresthesia, the UN injection may be performed proximal to the retroepicondylar groove (Supplementary Video 3). To confirm the needle tip's epineural position, a test injection with lidocaine can be performed to see the injectant's epineural flow. To provide total coverage of the injectate around the nerve, it is sometimes necessary to reposition the needle to the other side of the nerve. This hydrodissection separating the UN from the ME end might be followed by US during the injection (85). According to a recent randomized controlled trial (RCT), the effect of dextrose injection was superior to that of steroid injection (89). vanVeen et al. (90) in their study used visualization in the long axis, which, according to other authors, is less convenient because the nerve can be confused with other structures (91). In a case report, Stoddard suggested that hydrodissection with a higher injected volume might also be beneficial (92).

A recent systematic review evaluating conservative treatment of cubital tunnel syndrome proposed that steroid injection decreased nerve CSA. The review's limitation was the paucity and heterogeneity of the studies concerning steroid or local anesthetic injection (61). Hong et al. (66) compared two conservative treatment approaches—splinting vs. splinting plus injection with corticosteroids and local anesthetic. A total of 10 patients (12 nerves) were assessed. Clinical evaluation and nerve conduction studies were performed 1 and 6 months after the intervention. Their results showed significant improvement in both groups' symptoms, and there were no significant differences between the two intervention groups. Therefore, splinting alone was concluded to be sufficient with no need for an additional steroid injection. However, the injections in this study were landmark-guided (66). vanVeen et al. (90) conducted a randomized, double-blinded trial to compare the effect of steroid injection with that of placebo injection. In total, 55 patients were involved in this study. The primary outcome was a subjective change in symptoms after 3 months from intervention. Secondary outcomes were CSA of the nerve and electrodiagnostic studies. The results showed that 30% of steroid group participants reported a favorable outcome, compared with 28% in the placebo control group. There was a significant decrease of CSA in the steroid injection group and no significant improvement in electrodiagnostic studies. The study concluded that the positive effect of US-guided steroid injection compared with placebo was not demonstrated (90). Rampen et al. (93) published a case series of seven patients with UNE, treated with steroid injection. Four out of seven patients reported clinical improvement (in terms of symptom relief and neurologic improvement) and CSA reduction 6 weeks following the intervention. Symptoms were unchanged in two of the patients and worsened in one patient. This study, however, lacked a control group, and the patients opted for injection because they disapproved of surgical treatment after initial conservative therapy failed (93). Alblas et al. (94) conducted a feasibility study with eight patients (nine UNEs) regarding US-guided steroid injection. During 3 months of follow-up, five patients reported improved symptoms, whereas three patients had no change in symptoms, and one patient reported worsening of the symptoms. The study concluded that US-guided steroid injection was as safe and easy (94).

Another feasibility study was conducted by Choi et al., (88) who assessed the in-plane approach of US-guided steroid injection for cubital tunnel syndrome in 10 patients. Their results showed a statistically significant decrease in the severity of the symptoms as evaluated by the VAS and CSA decrease in the first and fourth week of follow-up. No side effects were reported (88). A recent RCT by Chen compared the effect of steroid injection with that of dextrose injection in patients with UNE. In total, 33 patients completed the study. The primary outcome was digital pain/paresthesia evaluated with VAS. Secondary outcomes were disability questionnaires, nerve conduction studies, and CSA of the UN. There was a more considerable decrease in symptom severity in the dextrose group from the third month of follow-up and onward. The study concluded dextrose to be more suitable for perineural injection in patients with UNE (89).

## Surgical Treatment

In 1957, Osborne described the first series of surgically treated patients with spontaneous UNE (95). Surgical treatment of cubital tunnel syndrome remains controversial. Although many techniques may be used for decompressing the UN, there are no clear consensus for one approach over another. This uncertainty was not resolved even by several systematic reviews published during the last decade. Therefore, the choice of approach is often based on personal experience and subjective preference for specific clinical findings. Almost 90% of surgeons use more than one procedure in the treatment of cubital tunnel syndrome (96). However, up to 30% of the patients do not improve after surgery and require revision procedures, which is even more controversial and rarely curative (97, 98).

### *In situ* Decompression

**Figure 11:**
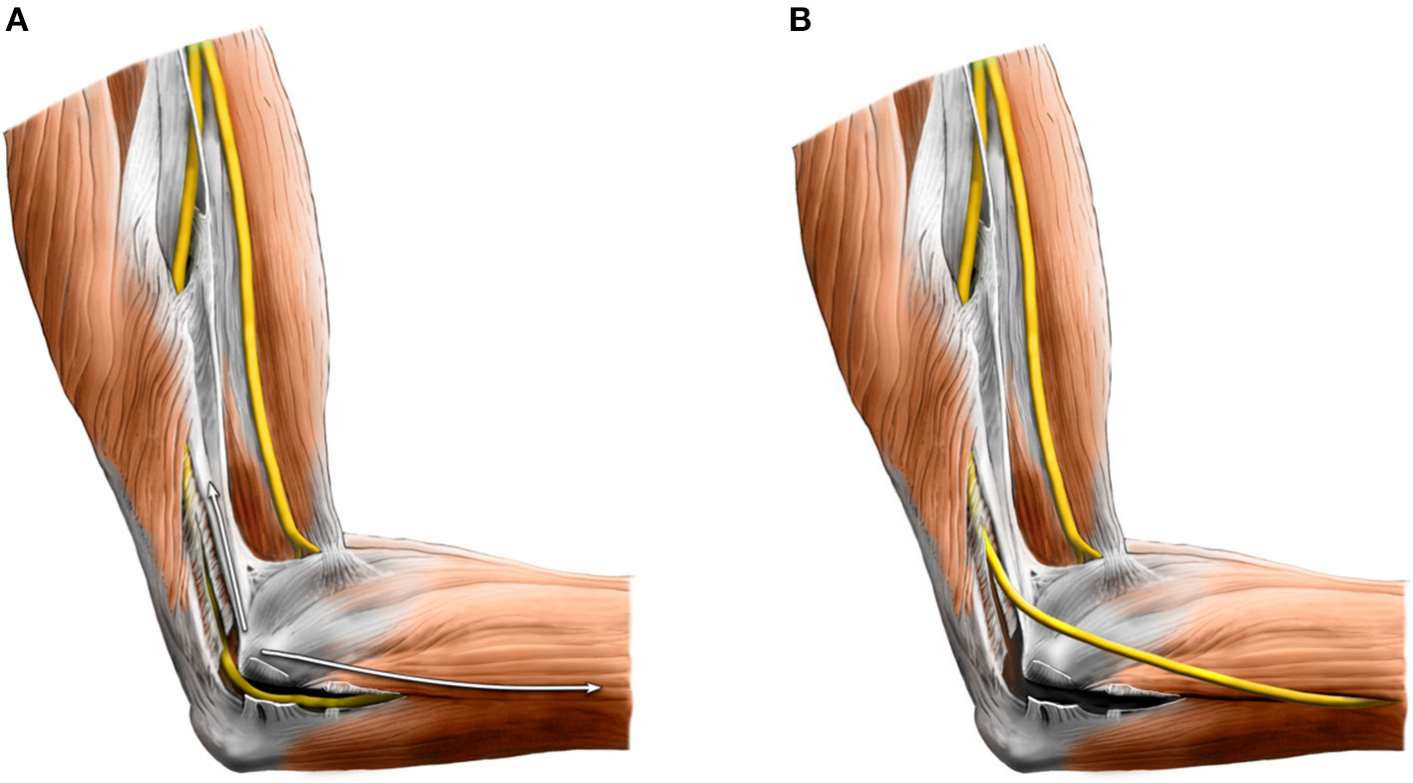
**(A)** *In situ* decompression of the ulnar nerve in the cubital tunnel. **(B)** Anterior subcutaneous transposition—after wide decompression, the ulnar nerve is transposed anteriorly under the cutaneous flap.

Simple decompression is a basic and probably the most commonly used technique, particularly beneficial when nerve entrapment is the underlying cause of UNE. It is easy to perform and generally free of complications. It is performed from a small incision above the ME parallel to the course of the UN. Care must be taken to protect the posterior branches of the medial antebrachial cutaneous nerve. The surgery aims to release the nerve by cutting the superficial fascia of the FCU muscle, retroepicondylar groove retinaculum, and the HUA. However, it is always necessary to explore the nerve proximally toward the MIS of the arm to check for any compression by the arcade of Struthers or by the septum itself. Similarly, the nerve is explored distally to the proximal forearm to release possible compression within the FCU by the thicker parts of the intermuscular connective tissue (Figure 11A). After sufficient decompression of the nerve, flexion and extension of the elbow are examined to rule out subluxation over the ME (99).

In such cases, the decompression can be facilitated by the medial epicondylectomy, which allows a mini-anterior transposition without excessive dissection and devascularization of the nerve. It is recommended to remove less than 4 mm of the ME's width in the coronal plane to prevent damage of the anterior part of the medial collateral ligament, which may result in elbow instability or medial elbow pain (100). Some authors, however, prefer to perform an anterior transposition of the nerve to preclude chronic injury to the nerve by its repetitive subluxation (101).

### Anterior Transposition

Transpositional surgical treatment may be performed by subcutaneous, intramuscular, and submuscular techniques. The transposition aims to reduce the tension on the nerve and prevent further compression in the cubital tunnel by bony spurs, synovial swelling, or chronic subluxation (102).

All other techniques than *in situ* decompression require a longer incision (~6 cm). The nerve is transposed anteriorly under the skin flap (or intra or under the forearm flexors' common head) after its wide release from the original bed. The easiest and most commonly performed technique is subcutaneous transposition (Figure 11B). Submuscular or intramuscular transpositions are much more invasive and, therefore, carried out less frequently, especially in patients with minimal amounts of subcutaneous fat or in some revision cases. The argument for higher invasiveness is to create a healthy vascular bed protected by soft tissue. Nevertheless, Liu et al. (101) found in their meta-analysis that subcutaneous and submuscular transpositions are equally effective.

Said et al. (103) demonstrated in their meta-analysis no difference in outcome or revision rate between simple decompression and anterior transpositions in primary cubital tunnel syndrome. Similarly, Chen et al. (102) found the same effect of both methods and a significantly lower incidence of complications in cases operated by simple decompression. Anterior transposition is often used in revision release after failed primary decompression. Moreover, some authors recommend submuscular transposition after failed subcutaneous transposition (104). However, there is no robust evidence supporting the need for anterior transposition in recurrent cubital tunnel syndrome (105).

Moreover, an excessive release of the nerve before its transposition is associated with decreased regional blood flow to the nerve for at least 3 days. Those mentioned above may increase the complication rate after surgery (102).

### Endoscopic Decompression

The endoscopic technique was introduced as a minimally invasive alternative for open decompression, aiming to minimize trauma to the tissues and improve postoperative recovery. Its theoretical advantages are the patient's faster recovery, decreased invasiveness, minimal adverse events, and less scar discomfort. However, it should be applied only in selected cases without evidence of nerve subluxation, traumatic etiology of cubital tunnel syndrome, or significant structural pathology (106).

Schmidt et al. (107) and Krejčí et al. (99) performed RCTs comparing open and endoscopic decompression. In both studies, the authors failed to show any additional benefit of the endoscopic technique, and they concluded that both techniques are equally effective. These results were in line with several systematic reviews and meta-analyses (106, 108, 109).

However, it has been proven that endoscopic technique is associated with a lower incidence of scar tenderness or elbow pain (106). Moreover, it is performed with a smaller skin incision (1.5–2 cm) compared with open decompression (~4 cm). On the other hand, it is significantly longer than open surgery. Although the difference in the median duration of decompression (i.e., incision to suture time) was only 6 min in a study of Krejčí et al. (99) (30 min for open and 36 min for endoscopic techniques, respectively), the setup time was almost three times longer in the endoscopic procedure (6 and 18 min, respectively). Another disadvantage is that it is necessary to have an assistant holding the arm in place and changing the flexion degree as needed (99).

### Summary of the Techniques

Wade et al., (98) in 2020, performed a comprehensive review and meta-analysis of all possible open or endoscopic methods for treating cubital tunnel syndrome. They found that open *in situ* decompression (with or without medial epicondylectomy) appears to be the safest and most effective method for primary cubital tunnel syndrome patients. It was associated with the greatest response to treatment and the lowest risk of complications, reoperation, and recurrence. They also showed that *in situ* decompression (open, minimally invasive, or endoscopic) was associated with a lower risk of complications than any form of transposition. Moreover, the addition of epicondylectomy led to a higher success rate without increasing the risk of complications. Another advantage of *in situ* decompression is the reduced operative time and its simplicity. Of note also is that it is 18–55% cheaper than the transposition procedure (98). Therefore, open *in situ* decompression should be considered a first choice in treating patients with primary cubital tunnel syndrome. In recurrent cases, the surgeon should consider the extent of primary decompression, previous elbow trauma, and possible chronic subluxation to decide whether to perform more extensive decompression only or an anterior transposition. To this end, one should plan the surgery concerning local anatomy (e.g., anatomic variations and space-occupying lesions), where US can provide valuable information preceding the surgery, e.g., aberrant vein (Figure 9F).

## Conclusions and Future Directions

Cubital tunnel syndrome is commonly encountered in daily clinical practice. If correctly diagnosed, the treatment outcome can be promising. In the light of the broad differential diagnosis, a convenient imaging tool may be necessary in some cases. Hence, high-resolution US can be an inexpensive, safe, and accessible modality for visualizing and guiding the treatment of UN neuropathy around the elbow. US imaging in such indications can be expected to increase its awareness among physicians worldwide in the near future.

## Author Contributions

KM devised the project and the main conceptual ideas. ON supervised this work. Five authors wrote the first drafts of the manuscript sections (KM—introduction and US assessment. JJ—US-guided procedures and anatomy. RK—surgical techniques. SM—non-operative treatment. ON—anatomy). JJ formatted the figures and their legends. ON and KM dissected the cadaveric specimen and obtained the corresponding photographs. PS provided exemplary US images together with figure legends. KS, YA, and PS (together with other co-authors) provided critical feedback, helped shape the manuscript, and obtained figures and videos. All authors approved the final manuscript.

## Conflict of Interest

The authors declare that the research was conducted in the absence of any commercial or financial relationships that could be construed as a potential conflict of interest.

## Abbreviations

CSA: cross-sectional area
FCU: flexor carpi ulnaris
ME: medial epicondyle of the humerus
MIS: medial intermuscular septum
RCT: randomized controlled trial
UN: ulnar nerve
US: ultrasound.
